# Diabetic Coma

**Published:** 1891-05-23

**Authors:** 


### DIABETIC COMA.

What are the causes of diabetic coma? and How should we treat it? are two questions that hitherto have found no very satisfactory answer.

It has been supposed that the immediate cause of diabetic coma was acetonæmia, the action of aceton having an effect very similar to that of ether and alcohol, though not quite so powerful. But the amount of aceton circulating in the blood is not sufficient to account for the symptoms; moreover, as the comatose condition comes on, the aceton is not infrequently diminished, its less oxidised predecessor, oxybutyric acid, being more abundant, the latter having no narcotic action. Minkowski's and Stadelmann's explanation of this is that the blood is unable to form the necessary loose chemical combination with the carbonic acid in the tissues, owing to its alkaline salts being fixed by oxybutyric acid and other acids resulting from incomplete oxidation of proteids. Thus the carbonic acid is not conveyed by the blood to the lungs and eliminated. Dr. Schmitz has recently published his views as to the nature and causes of diabetic coma.*

Dr. Schmitz recognises two distinct causes. The first is weakness of the heart's action, caused by the effect of the sugar in the blood on the muscular fibres of the heart. In diabetic subjects, even when fat and seemingly in good condition, the body muscles are lax and soft, and the results are feeble gait and weariness easily produced. The heart muscle, he argues (and post-mortems support him), is in a similar condition, also suffering from degeneration of its fibres. The cyanotic face, the quick breathing, and the slow, small pulse, getting quick and irregular with the slightest exertion, point in that direction. Then the cardiac impulse is hardly perceptible, the area of the heart's dulness is increased in breadth, and, on auscultation, not only at the apex, but on the aorta, the first sound is anything but clear. Suppose such a heart is wearied out by over-exertion, the result is that the breathing is affected, the appetite disappears, the head suffers from giddiness, pain, and an inclination to vomit, and if no precautions are taken, or prove of no avail, the arteries get more and more empty, the veins more and more full, and carbonic acid accumulates in the blood, somnolence steals on, coma succeeds, and the patient dies poisoned. In such cases the line of treatment is plain. Anything exciting the heart, medicinal or other, must be avoided. Hill climbing, stair climbing, long walks, early rising, Venus and Bacchus in excess are all to be avoided, as also are bromide of potassium, all combinations of potassium, salicyl, antipyrin, antifebrin, and hot baths. Also narcotics, except when absolutely necessary, and then carefully used. Nutritious, but easily digested food, a moderate amount of alcohol, fresh and invigorating air—these are the best drugs. Of course, one must not lose sight of the ordinary diabetic treatment; the above refers to cardiac failure. In a pressing case the patient is to be kept in the recumbent position, and must neither stand up nor sit up, even for stools and urinating. Stimulants by the mouth, or, preferably, if apt to cause vomiting, subcutaneously (*e.g.*, musk and camphor). The best stimulant of all is black coffee. With alcohol in large doses it is necessary to be careful, on account of the succeeding depression favouring collapse. The neglected *castoreum sibiricum*, not *canadense*, he considers a very good stimulant. The great danger lies in letting the patients up too soon, and the great difficulty is keeping them in bed long enough.

The second form of diabetic coma he describes as an acute self-poisoning, which has been named, although not correctly, acetonœmia. This condition is preceded by symptoms mistaken for indigestion, or slight gastric catarrh, with great weariness and sleepiness. The sleep, like that of drunkenness, leaves the patient more weary, feeble, and heavy-headed than before it. There is usually constipation, sometimes diarrhœa, the tongue coated and dry, and the breath smells badly. In about two days he becomes so drowsy that it requires a loud call to rouse him; he seems peevish, and hardly answers a question, and quickly relapses into his sleepy condition. He complains of colicky pains in the abdomen, which become very severe. After violent efforts he vomits some green-coloured fluid, the pain is then somewhat relieved, and he falls asleep until another attack.

The heart and lungs on examination reveal nothing abnormal. The pupils are generally contracted, and react but slightly. Towards the end violent clonic and tonic spasms set in, and death follows in deep coma. The colic, the high temperature, and the clear heart sounds distinguish this form of coma from that produced by cardiac failure. The treatment consists in clearing out the bowels, constipated or not, with one or two tablespoonfuls of castor oil. "The poison, be it ptomaïne or toxine, or whatever else, is a product of decomposition in the bowel." After a thorough purging, resulting in a profusion of black, foul, fœtid stools, recovery is remarkably quick. Of eight cases, all equally bad, four not treated with the oil died, and four treated with it speedily recovered.

The clinical success reported by Dr. Schmitz go a long way to support his views as to treatment, but a large number of cases are unfortunately always fatal in spite of all treatment. Dr. Saundby† reports that in a case under his care the inhalation of oxygen was tried without success, while the subcutaneous injection of strychnine and intraperitoneal injection of an alkaline solution also failed.

* Schmitz, *Berl. Klin. Woch.*, Aug. 25th, 1890; Stewart, *Med. Chron.*, Nov., 1890.

† Saundby, *Medical Annual*, 1890.

